# Sharing clinical and forensic toxicology knowledge in the era of a pandemic and beyond

**DOI:** 10.1080/20961790.2021.1997081

**Published:** 2021-11-19

**Authors:** Nikolas P. Lemos

**Affiliations:** Cameron Forensic Medical Sciences, William Harvey Research Institute, Barts and The London School of Medicine and Dentistry, Queen Mary University of London

Science matters, especially in times of crisis. As history clearly shows, we often rely on science to help solve some of the most pressing technical challenges and identify solutions that address major public health crisis including the Spanish flu, polio, and HIV/AIDS. In the ongoing struggle to safeguard humankind throughout time, science has been a reliable constant. This year, as the world faced yet another challenge, scientists mobilized in unprecedented ways through global collaborations and innovative technologies, to bring to every village, tribe, town, and city of the world the best chance of beating COVID-19. On December 14, 2020 as the first batch of a federally approved coronavirus vaccines in the USA were administered to health care workers across that country, science emerged victorious once again.

Just like in the case of COVID-19, the issues and challenges that analytical, clinical and forensic toxicologists face on a daily basis in their own local communities around the world need timely and accurate solutions.

The International Alliance of Clinical and Forensic Toxicologists (IACFT) is a global toxicology collective which strives to offer these solutions in an affordable (free) and safe (virtual) environment. The issue of *Forensic Sciences Research* you are reading is a brief representation of the work presented during IACFT’s inaugural meeting in late 2020. The meeting was held in November 2020 and was free for all, virtual, and multilingual with presentations simultaneously in Arabic, Chinese, English, French, Portuguese, Spanish and Turkish. The entire event was a very humbling experience given that hundreds of colleagues from every part of the world were virtually present from the safety and comfort of their own office or home for what proved to be a very exciting and revolutionary way of sharing knowledge. Many of those who participated in IACFT’s virtual inaugural meeting have worked in the field of clinical and/or forensic toxicology for years and have previously participated in the traditional sharing of knowledge through The International Association of Forensic Toxicologists (TIAFT), Society of Forensic Toxicologists (SOFT) and the American Academy of Forensic Sciences. However, nobody could resist the opportunity to join the virtual celebration of toxicology that IACFT offered for free without any risk of contracting COVID-19 and without any associated expense to do so.

Our Founding Members met on April 15, 2020 to brainstorm about ways to continue sharing knowledge, exchanging ideas, and bringing scientists and students closer who for reasons such as the pandemic-related lockdowns financial restrains, difficulties with leaving work or home, and language limitations, could not participate in traditional professional continuing education. The Founding Members decided that IACFT would be the new, agile, virtual, multilingual and free forum by toxicologists — for toxicologists which would serve as a force for our science communications and exchange of knowledge to continue.

The Alliance recognized from the time of its inception the diversity of the world’s toxicologists and maked it part of its mission to foster growth of all that talent that we believed remained underappreciated by and underrepresented in existing professional groups. The Alliance pledged to invite everyone to the main table, to the main event, irrespective of financial ability to travel, spoken language or ability to pay membership or meeting registration fees. The Alliance’s driving principles include recognizing and respecting diversity, welcoming this diversity, and giving an equal voice to this diverse group of scientists.

In a nutshell, the Alliance understands that equal but separate is not equal.

The Alliance believes that costs and expenses associated with travel, hotel, taking time off work, paying for childcare, freight, and cargo expenses if you are a sponsor, or any other expenses associated with traveling to a face-to-face conference location, no matter how beautiful or exotic of a destination, should not be a limiting factor in the participations of toxicologists and students in continuing education. The Alliance recognizes how critical it is during times of pandemics and financial uncertainties to make continuing education and sharing of knowledge across continents, across borders and across languages, an easier, more affordable, and more inclusive activity.

Among those who recognized this incredible opportunity to be part of our revolution in the sharing of knowledge are:

- The Centre for Forensic Science Research and Education of the Fredric Reiders Family Foundation,
- The Association for Mass Spectrometry and Advances in the Clinical Lab,
- The Turkish Society of Clinical and Forensic Toxicology, and
- The clinical toxicology working group of the Turkish Pharmacology Society.

Additionally, numerous academic institutions and universities support our global effort and give time and resources to their academic faculty and staff to work with us and to extend collaborative hands to scientists beyond their local communities:

- Ege University in Turkey,
- The Institute of Technology, Sligo in Ireland,
- Lincoln University in the USA,
- The University of California, San Francisco in the USA,
- Queen Mary University of London in England, and
- The National and Kapodistrian University of Athens in Greece.

But more than anyone else, we wish to acknowledge *Forensic Sciences Research*. The open access journal has been on the Alliance’s side since the beginning and has been a key driving force for our members and conference participants to excel and to always aim higher. The work you are about to read comes from all over the world, including from members of our invited partner association during our inaugural event, the Algerian Society of Toxicology. It is a true representation of a global force; a global wave of change.

The articles that are included in this issue are a small fraction of what was presented at the virtual meeting but offer a wonderful glimpse into our community’s abilities and potential. You will read about the newest and latest developments on scientific methods, innovations and approaches that have been optimized and validated by scientists whose efforts to expand and share knowledge are represented in the following pages. The scientific programme of our inaugural virtual meeting reflects our global community’s best efforts in detecting and restricting intoxicants and drugs in many aspects of our daily lives including sport, work, travel, and driving to name just a few, and draws from the expertise of local experts with global reach. Prepare yourself to be amazed by our community’s methodologies and techniques which permit the detection of an ever-increasing number of substances at ever-decreasing concentrations in a variety of biological specimens and types of cases including postmortem, driving and alleged doping and drug facilitated crimes.

This issue is the result of the hard work of all the clinical toxicologists, forensic toxicologists, students, translators and administrators who volunteered their talent and time to make IACFT a reality and to make our inaugural meeting such an educational collection of knowledge.

Please be safe and healthy and please consider joining the Alliance and becoming part of the online revolution at www.iacft.online.


Nikolas P. Lemos
*Cameron Forensic Medical Sciences, William Harvey Research Institute, Barts and The London School of Medicine and Dentistry, Queen Mary University of London, London, UK*

n.lemos@qmul.ac.uk



